# Molecular Tools for Early Detection of Invasive Malaria Vector *Anopheles stephensi* Mosquitoes

**DOI:** 10.3201/eid2901.220786

**Published:** 2023-01

**Authors:** Om P. Singh, Taranjeet Kaur, Gunjan Sharma, Madhavinadha P. Kona, Shobhna Mishra, Neera Kapoor, Prashant K. Mallick

**Affiliations:** National Institute of Malaria Research, New Delhi, India (O.P. Singh, T. Kaur, G. Sharma, M.P. Kona, S. Mishra, P.K. Mallick);; Indira Gandhi National Open University, New Delhi (N. Kapoor)

**Keywords:** Anopheles stephensi, malaria, vector, invasive species, PCR, ITS2, vector-borne infections, mosquitoes, India

## Abstract

Reports of the expansion of the Asia malaria vector *Anopheles stephensi* mosquito into new geographic areas are increasing, which poses a threat to the elimination of urban malaria. Efficient surveillance of this vector in affected areas and early detection in new geographic areas is key to containing and controlling this species. To overcome the practical difficulties associated with the morphological identification of immature stages and adults of *An. stephensi* mosquitoes, we developed a species-specific PCR and a real-time PCR targeting a unique segment of the second internal transcribed spacer lacking homology to any other organism. Both PCRs can be used to identify *An. stephensi* mosquitoes individually or in pooled samples of mixed species, including when present in extremely low proportions (1:500). This study also reports a method for selective amplification and sequencing of partial ribosomal DNA from *An. stephensi* mosquitoes for their confirmation in pooled samples of mixed species.

Reports of the expansion of the malaria vector *Anopheles stephensi* mosquito (*1*–*6*) are increasing, intensifying the threat of urban malaria (*7*). The gradual southward expansion of this species has been recorded in India since the 1970s (*1*). The first occurrence of *An. stephensi* mosquito in Africa was reported as early as 1966 in a town in Egypt (*2*). During the past 2 decades, several reports of expansion of this species in the Horn of Africa, Sudan, Sri Lanka, and Lakshadweep (a union territory of India) have appeared (*1*–*6*). A high probability of presence within many urban cities across Africa has been predicted, which warrants prioritizing vector surveillance (*8*). 

As a consequence of recent invasions of this vector species in several countries, the World Health Organization (WHO) recommended conducting active surveillance of *An. stephensi* mosquitoes in urban and periurban areas, in addition to routine surveillance in rural areas in the affected and surrounding geographic areas (*9*). However, identifying this species, a process that relies mainly on the morphologic characteristics of adult female mosquitoes, is often challenging. Sampling of adult *An. stephensi* mosquitoes from their resting habitats is difficult because they are secretive in their habits (*10*). WHO has recommended sampling immature *An. stephensi* mosquitoes from natural breeding habitats and rearing them in the laboratory until their emergence into adults to enable species identification (*9*). This practice is being adopted for sampling this species in regions of Africa (*4*,*5*,*11*), which is a time-consuming and labor-intensive process. Even freshly emerged adults have been reported to be misidentified as *An. gambiae* mosquitoes because of superficial resemblance (*11*). 

Adult mosquito collection through light-trap or pyrethrum spray collections are alternative and popular methods of sampling *An. stephensi* mosquitoes, but identifying adults collected through such methods can be difficult because of the loss of morphologic characteristics critical for their correct identification. Therefore, developing highly specific PCR-based assays is crucial for identifying both larval and adult *An. stephensi* species collected by a variety of methods. Such diagnostics will be helpful to field technologists who are not familiar with the morphology of this species. Those assays can detect *An. stephensi* mosquitoes in a large pool of mosquitoes when their proportion is extremely low. Equally vital is developing a DNA sequencing strategy to confirm the presence of *An. stephensi* mosquitoes in such pools of mixed species. Such molecular tools will help detect invasions of *An. stephensi* mosquitoes early in new geographic areas where they are present in extremely low densities.

## Methods

### Mosquito Samples

We obtained adult mosquitoes or their DNA samples belonging to a total of 17 anopheline and 3 culicine species from different parts of the world either from BEI Resources (https://www.beiresources.org) or locally (Appendix Table). Field-collected *An. stephensi* mosquitoes were processed for identifying biologic forms according to the methods described by Singh et al. (*12*).

### DNA Isolation from Individual and Pooled Samples

We isolated DNA from individual specimens as well as pooled samples of mosquitoes to standardize PCR-based assays and their validation. We used pooled samples of different sizes, each consisting of a single *An. stephensi* mosquito and the rest *An. culicifacies* mosquitoes*.* We also used a pool of field-collected mosquitoes consisting of a single *An. stephensi* mosquito and other species.

We selected 2 commercial kits for DNA isolation. For DNA isolation from individual mosquitoes or smaller pool sizes, we used the DNeasy Blood and Tissue kit (QIAGEN, https://www.qiagen.com), recommended for >25 mg of tissue. For larger pools, we used DNAzol Reagent (ThermoFisher Scientific, https://www.thermofisher.com), recommended for 25–50 mg of tissue per milliliter of reagent. In addition, we isolated DNA from individual *An. stephensi* mosquitoes by boiling method.

#### DNeasy Blood and Tissue Kits

We isolated DNA from individual mosquitoes of some *Anopheles*, *Culex*, and *Aedes* species (Appendix Table) following the manufacturer’s protocol and eluted in 200 µL elution buffer. We isolated DNA from smaller pools of III–IV instar larvae or adults, each containing a single *An. stephensi* mosquito and the rest *An. culicifacies* mosquitoes in different pool sizes (i.e., 2, 5, 10, 20, and 50). We also isolated DNA from the single leg of an *An. stephensi* mosquito, which was eluted in 50 µL of elution buffer.

#### DNAzol Reagent

We used DNAzol Reagent for DNA isolation from pools (i.e., 25, 100, and 500) of adult mosquitoes and pools of 100 larvae (III–IV instar), each containing 1 *An. stephensi* mosquito and the rest *An. culicifacies* mosquitoes. We directly homogenized pools of 25 and 100 mosquitoes in 1 mL of DNAzol reagent in a microcentrifuge tube. In the case of pools of 100 mosquitoes, we transferred 250 µL of the triturate to a separate microcentrifuge tube to make up a volume of 1 mL with DNAzol. We ground the pools of 500 mosquitoes in liquid nitrogen and transferred ≈25 mg of triturate in a microcentrifuge tube and homogenized in 1 mL of DNAzol reagent. We centrifuged all triturates at 10,000 × *g* for 10 min and transferred 500 µL of supernatant to a fresh 1.5-mL microcentrifuge tube, which we subjected to ethanol precipitation, washing, and solubilization of DNA following the vendor’s protocol. We dissolved DNA in 200 µL of 8.0 mM NaOH. We also isolated DNA from a pool of 100 mosquitoes containing a single *An. stephensi* mosquito and field-collected (through hand-catch method) mosquitoes belonging to *An. culicifacies*, *An. subpictus*, *An. fluviatilis*, and *Culex quinquefasciatus.*

#### Boiling Method

We isolated DNA from 10 individual specimens of *An. stephensi* mosquitoes by the boiling method described by Sharma et al. (*13*). We either used the DNA isolated from this method immediately after isolation or preserved it at –20 °C for later use.

### Selecting Target Sites for Designing Primers and Probes

**Figure 1:**
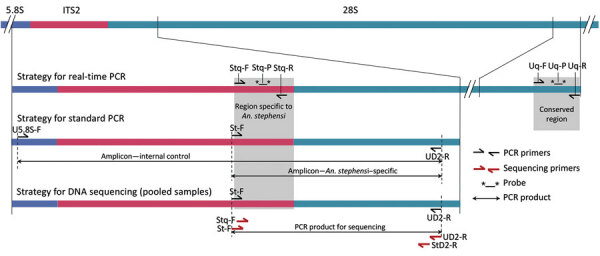
Schematic representation of PCR and sequencing strategies used for early detection of invasive malaria vector *Anopheles stephensi* mosquitoes

We selected ribosomal DNA (rDNA) as a target site for developing diagnostics to identify *An. stephensi* mosquitoes, which are present in hundreds of copies in an individual and are highly conserved in a species because of homogenization of sequence through unequal crossing over and gene conversion, a process known as concerted evolution. We selected the internal transcribed spacer 2 (ITS2) rDNA, which is conserved within a species but highly variable across taxa, for designing *An. stephensi*–specific primers and probes. For designing *An. stephensi*–specific primers and probes, we conducted a homology search of ITS2 sequences of *An. stephensi* (*14*) through a nucleotide BLAST search (https://blast.ncbi.nlm.nih.gov/Blast.cgi). We optimized the search for blastn (somewhat similar sequences) and excluded the taxon *Anopheles stephensi* from the search. All 297 search returns belonged to the *Anopheles* mosquitoes, all belonging to the *Neocellia* series (subgenus *Cellia*); however, none of the returns showed homology to the last 122 bp segment of ITS2. We considered this region unique to *An. stephensi* mosquitoes and exploited this region for designing highly specific *An. stephensi*–specific primers and probes (Figure 1). For designing universal primers and a probe, we identified highly conserved regions from 5.8S and 28S rDNA (Figure 1) based on the alignment of sequences of anophelines available in the GenBank. To verify the specificity of each *An. stephensi*–specific primer and probe (Table 1), we performed a blastn search in silico, which ensured that none of the primers and probes matched rDNA sequences of any other mosquitoes.

**Table 1:** Sequences of primers and probes used in study of molecular tools for early detection of invasive malaria vector *Anopheles stephensi* mosquitoes

Name	Sequence, 5′ → 3′	Annealing specificity	Reference
Stq-F	TCTTTCCTCGCATCCAGTTG	*An. stephensi*	This study
Stq-R	CGGGAGAAGCGGTGATAAAT	*An. stephensi*	This study
Stq-P	/56-FAM/CGTGCTAAC/ZEN/CTCACTCACCCACAC/3IABKFQ/	*An. stephensi*	This study
Uq-F	GAGATTCCCTCTGTCCCTATCT	Universal	This study
Uq-R	AGCTCAACAGGGTCTTCTTTC	Universal	This study
Uq-P	/5HEX/TAGCGAAAC/ZEN/CACAGCCAAGGGAA/3IABKFQ/	Universal	This study
U5.8S-F	ATCACTCGGCTCATGGATCG	Universal	(*15*)
St-F	CGTATCTTTCCTCGCATCCA	*An. stephensi*	This study
UD2-R	GCACTATCAAGCAACACGACT	Universal	This study
StD2-R	GTCTGCCACCACAGTCCT	*An. stephensi*	This study

### Development of a Hydrolysis Real-Time PCR 

For *An. stephensi*–specific hydrolysis real-time PCR, we designed 2 oligonucleotide primers, (Stq-F and Stq-R) and a hydrolysis probe (Stq-P) from the *An. stephensi*–specific ITS2 region (Table 1; Figure 1). For internal control (IC), we designed primers (Uq-F and Uq-R) and a hydrolysis probe (Uq-P) from a region of 28S-rDNA conserved in anophelines (Table 1; Figure 1). We performed real-time PCR in 10 μL of reaction mixture containing 0.4 μM each of Uq-F, Uq-R and Stq-F; 0.5μM of Stq-R; 0.2 μM of each probe (Stq-P and Uq-P); 1X QuantiFast Multiplex PCR kit (QIAGEN); and 1 μL of template DNA in Bio-Rad CFX96 Touch Real-Time PCR Detection System (https://www.bio-rad.com). The cyclic conditions were predenaturation at 95°C for 5 min, followed by 35 cycles, each with denaturation at 95°C for 10 s and annealing/extension at 60°C for 30 s. We scored the number of cycles required for the fluorescent signal to cross the threshold (cycle threshold [Ct] values) by using the software CFX Maestro (Bio-Rad) (Appendix Figure 1).

**Figure 2:**
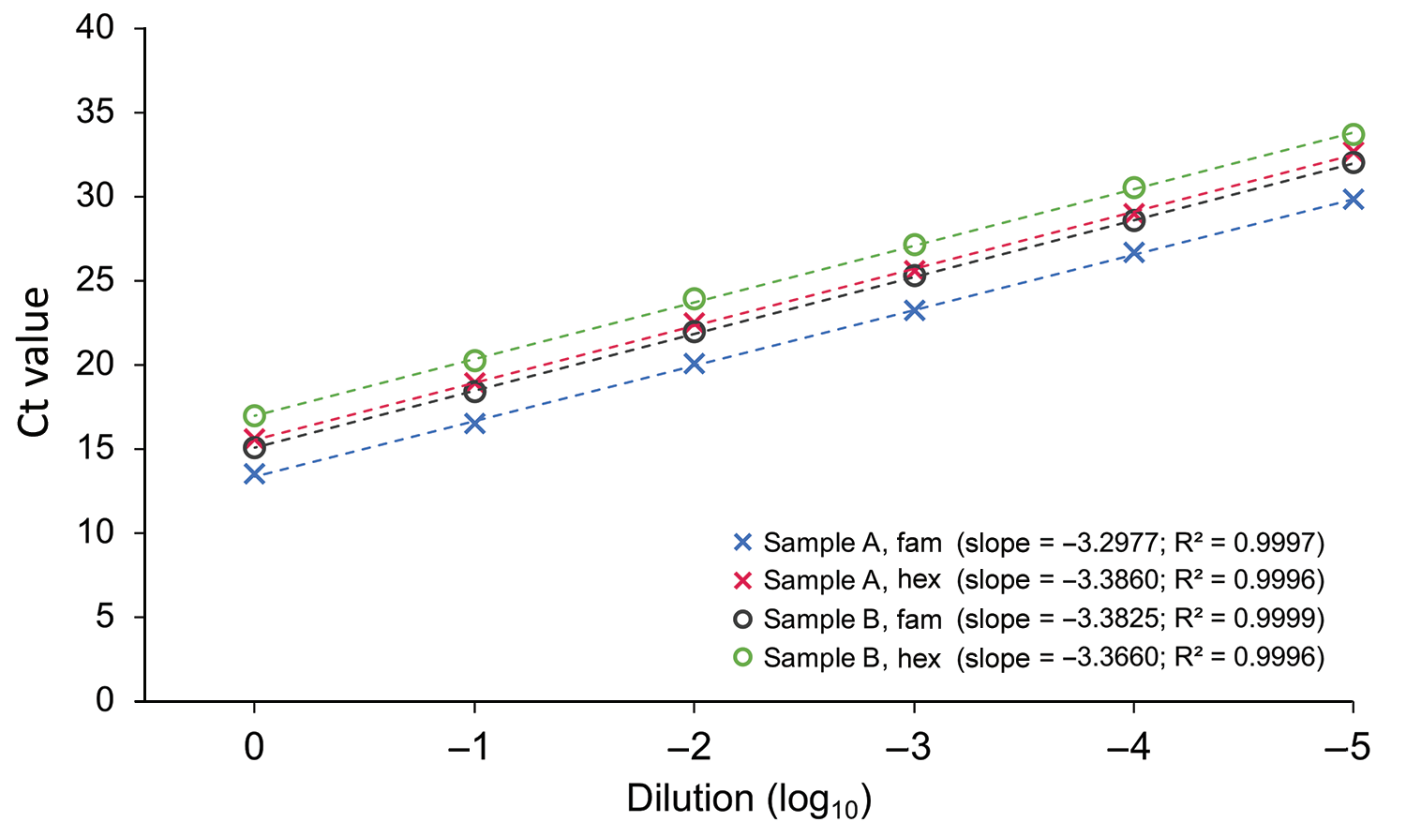
Standard curve showing correlation of Ct values against 10-fold serially diluted DNA samples of *Anopheles stephensi* mosquitoes (2 samples, A and B) in the duplex hydrolysis fluorescent probe assay. The slope of each line represents [–1/log_10_ (PCR efficiency)] for a hydrolysis probe assay. R^2^ represents correlation coefficient of a slope. Ct, cycle threshold.

We evaluated PCR efficiencies of each hydrolysis probe assay by performing duplex real-time PCR assays in triplicate at 6 different concentrations, diluted serially by 10-fold. We performed the experiments on 2 samples of *An. stephensi* DNA with different DNA concentrations, 8.7 and 3.2 ng/μL (Figure 2). To determine the limit of detection (LOD), we conducted real-time PCR on the diluted DNA of *An. stephensi* mosquitoes with concentrations of 160 fg, 80 fg, 40 fg, and 20 fg, each with 12 replicates.

### Development of Size-Diagnostic PCR

For a size-diagnostic PCR, we designed 3 primers: we designed the *An. stephensi*–specific forward primer (St-F) from the *An. stephensi*–specific region of ITS2 and designed 2 flanking universal primers from conserved 5.8S rDNA (U5.8S-F) and D2 domain of 28S rDNA (UD2-R). According to the strategy designed (Figure 1), an *An. stephensi*–specific diagnostic amplicon of 438 bp size will be formed by the primers St-F and D2-R, and a universal amplicon of varying sizes (>600 bp), depending upon the length of ITS2 in a particular species, will be formed by the primers 5.8S and D2-R. The universal amplicon will serve as an IC to rule out PCR failure.

Because of the competitive nature of primers in multiplex-PCR, we optimized 2 protocols of PCR on the basis of the number of mosquitoes in a pool. For individual mosquitoes or smaller pools, we conducted size-diagnostic PCR assays by using Hot Start Taq 2X Master Mix (New England Biolabs, https://www.neb.com) in a 20-µL reaction mixture containing 0.5 units of taq polymerase, 1.5 mM of MgCl_2_, 0.25 µM of primer St-F, 0.25 µM of primer U5.8S-F, 0.375 µM of UD2-R, and 0.50 µL of DNA template (protocol-1). In another protocol (protocol-2), we reduced the concentration of primer U5.8S-F to 0.10 µM and increased the concentration of UD2-R to 0.50 µM for larger pools of mosquitoes (25–500). We also observed that the intensity of amplicon in size-diagnostic PCR with pools of mosquitoes (>25) could be improved by the dilution of template DNA. Therefore, the template DNA of larger pools of mosquitoes (>25) was further diluted by 1/10 before using it as template DNA for size-diagnostic PCR reactions to minimize the PCR inhibitors in PCR reactions. However, such dilution was not required for real-time PCR. Optimized thermal cycling conditions were an initial denaturation step at 95°C for 30 s, 30 cycles each with a denaturation step at 95°C for 30 s, annealing at 55°C for 30 s and extension at 68°C for 45 s, and final extension at 68°C for 7 min. Five µL of PCR product was run on 2% agarose gel and visualized in the gel documentation system.

### DNA Sequencing Strategy for the Confirmation of PCR-Based Identification of *An. stephensi* Mosquitoes in Pooled Samples

We amplified *An. stephensi*–targeted amplicons from DNA isolated from pools of 100 (field-collected) and 500 mosquitoes, each pool containing a single *An. stephensi* mosquito, using primers St-F and UD2-R. We performed PCR by using Hot Start Taq 2X Master Mix in a 20 µL reaction mixture containing 0.25 μM of each primer. PCR conditions were similar to PCR protocol-1 but with extension time reduced to 30 s and number of cycles increased to 35. The amplified products were treated with Exo-Sap II, and sequence termination reactions were performed from both directions of strands using BigDye Terminator v3.1 Cycle Sequencing Kit (both ThermoFisher Scientific). The primers used for sequencing were the primers used for PCR amplification as well as the 2 internal primers (Stq-F and StD2-R) (Table 1; Figure 1). Both internal primers are specific to *An. stephensi* and were expected to provide a noise-free sequence by eliminating the possibility of sequencing nonspecific PCR product. The sequencing products were electrophoresed on an ABI Prism 3730xl (ThermoFisher Scientific).

## Results

### Real-time PCR

PCR efficiencies, as estimated based on Ct values of 6 serially diluted concentrations of DNA, were 97.5%–101% for *An. stephensi*–diagnostic (Fam-labeled probe) PCR and 97.4%–98.2% for IC (Hex-labeled probe) PCR (Figure 2). The dynamic range of Ct values for real-time PCR for *An. stephensi*–specific PCR was 13.5–32 and for IC-PCR was 15.5–33.5; the LOD was 40 fg of genomic DNA.

The real-time PCRs conducted on DNA isolated from individual *An. stephensi* mosquito samples, 19 different nontarget *Anopheles* mosquito species, and a single *An. stephensi* mosquito in different pool sizes were specific based on software-determined Ct value scored within 35 cycles of reactions, except in the case of 2 preisolated DNA (1 each of *Aedes aegypti* and *An. subpictus* mosquitoes) showing false positivity with late Ct values (>32) (Table 2). The 2 false-positive samples were contaminated with the *An. stephensi* mosquito DNA as revealed through *An. stephensi*–targeted sequencing (Appendix). Although primers and probe for IC were designed on the basis of anophelines’ 28S rDNA sequences, they also worked on all 3 nonanopheline species tested (i.e., *Cx. quinquefasciatus*, *Ae. aegypti*, and *Ae. albopictus* mosquitoes)*.* The real-time PCR also successfully identified *An. stephensi* mosquito from DNA samples isolated by boiling method and DNA isolated from a single leg of mosquitoes. The real-time PCR was sensitive to detecting a single *An. stephensi* mosquito in pools of 500 mosquitoes with low Ct values (<24) (Table 2). Rising of fluorescent signal was noticed with IC probe in some experiments only in negative controls after 30 cycles but not with *An. stephensi*–specific probe (Appendix Figure 1).

**Table 2:** Results of hydrolysis real-time PCR and size-diagnostic PCR on individual and pooled mosquitoes for early detection of invasive malaria vector *Anopheles stephensi* mosquitoes*

Specimen type	DNA isolation method	Real-time PCR				Size-diagnostic PCR		
		No.	Ct values (*An. stephensi*)†	Ct values IC		No.	*An. stephensi*– specific band	IC band
Anophelines								
*Neocellia* series								
*An. stephensi* type	DNeasy	26	13.47–17.23	15.20–18.93		24	Positive	Positive
*An. stephensi* intermediate	DNeasy	2	14.75–15.60	15.39–16.06		2	Positive	Positive
*An. stephensi* var. *mysorensis*	DNeasy	1	14.54	15.01		1	Positive	Positive
*An. stephensi* strain STE2	DNeasy	1	14.85–15.25	16.06–16.51		2	Positive	Positive
*An. stephensi* type form	Boiling	10	15.17–17.41	15.97–18.05		-	Not done	Not done
*An. stephensi* type form, single leg	DNeasy	4	18.64–22.29	19.02–23.51		4	Positive	Positive
*Pyretophorus* series								
* An. gambiae*	DNeasy	3	Negative	14.58–17.32		3	Negative	Positive
* An. quadrimaculatus*	DNeasy	2	Negative	14.52–15.64		2	Negative	Positive
* An. merus*	DNeasy	2	Negative	15.22–15.35		2	Negative	Positive
*An. subpictus* form A	DNeasy	2	Negative	16.61–17.02		1	Negative	Positive
*An. subpictus* form A‡	Pre-isolated§	1	33.09	18.32			Negative	Positive
*An. subpictus* form B	Pre-isolated§	2	Negative	16.17–16.35		1	Negative	Positive
*An. sundaicus* cytoform D	Pre-isolated§	1	Negative	14.47		1	Negative	Positive
*Albimanus* series								
* An. albimanus*	DNeasy	2	Negative	16.21–16.58		1	Negative	Positive
*Anopheles* series								
* An. freeborni*	DNeasy	2	Negative	15.73–15.81		1	Negative	Positive
* An. atroparvus*	DNeasy	2	Negative	14.70–15.88		1	Negative	Positive
*Neomyzomyia* series								
* An. dirus*	DNeasy	2	Negative	17.34–18.01		1	Negative	Positive
* An. farauti*	DNeasy	2	Negative	19.09–19.48		1	Negative	Positive
*Myzomyia* series								
* An. funestus*	DNeasy	2	Negative	15.50–15.62		1	Negative	Positive
*An. culicifacies* species A	DNeasy	1	Negative	17.41		1	Negative	Positive
*An. culicifacies* species B	Pre-isolated§	1	Negative	17.48		1	Negative	Positive
*An. fluviatilis* species S	Pre-isolated§	1	Negative	16.61		1	Negative	Positive
*An. fluviatilis* species T	Pre-isolated§	1	Negative	17.02		1	Negative	Positive
Culicines								
* Culex quinquefasciatus*	DNeasy	3	Negative	16.58–19.20		2	Negative	Positive
* Aedes albopictus*	DNeasy	2	Negative	16.65–16.68		2	Negative	Positive
* Ae. aegypti*	DNeasy	2	Negative	18.96–19.20		2	Negative	Positive
*Ae. aegypti*‡	Pre-isolated§	1	32.95	20.97			Negative	Positive
Single *An. stephensi* individual pooled with *An. culicifacies*¶								
Adults (1/2)	DNeasy	1	17.06	18.31		1	Positive	Positive
Adults (1/5)	DNeasy	1	15.37	16.11		1	Positive	Positive
Adults (1/10)	DNeasy	1	15.77	15.30		1	Positive	Positive
Adults (1/20)	DNeasy	1	15.74	13.71		1	Positive	Positive
Adults (1/50)	DNeasy	1	15.43	11.43		1	Positive	Positive
Adults (1/25)	DNAzol	1	14.53	12.61		1	Positive	Positive
Adults (1/100)	DNAzol	1	16.37	11.27		1	Positive	Positive
Adults (1/500)	DNAzol	2	20.12–23.96	11.08–11.50		2	Positive	Positive
Larvae (1/2)	DNeasy	1	15.56	16.87		1	Positive	Positive
Larvae (1/5)	DNeasy	1	15.49	16.65		1	Positive	Positive
Larvae (1/10)	DNeasy	1	16.36	15.53		1	Positive	Positive
Larvae (1/20)	DNeasy	1	16.51	15.06		1	Positive	Positive
Larvae (1/50)	DNeasy	1	16.27	14.40		1	Positive	Positive
Larvae (1/100)	DNAzol	1	18.14	13.16		1	Positive	Positive
Single *An. stephensi* individual pooled with field collected mosquitoes (mixed mosquito-species)¶								
Adults (1/100)	DNAzol	1	17.61	11.70		1	Positive	Positive

*Ct, cycle threshold; IC, internal control. †Ct value shown as Negative implies that no Ct value was scored within 35 cycles of reactions. ‡Contaminated with *An. stephensi* DNA. §Preserved DNA isolated manually for other studies. ¶Denominator of numeric expression inside parenthesis indicate total number of mosquitoes in a pool.

### Size-Diagnostic PCR

**Figure 3:**
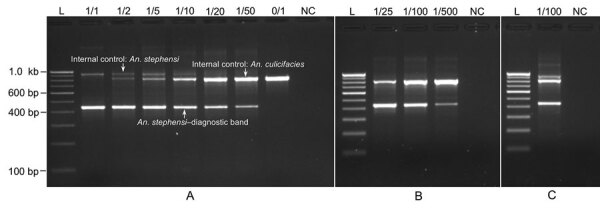
Gel photographs visualizing the result of PCRs specific to invasive malaria vector *Anopheles stephensi* mosquitoes. A) PCR protocol 1 on individual specimens and pools of *An. stephensi* and *An. culicifacies.* B) PCR protocol 2 on larger pools of *An. stephensi* and *An. culicifacies*. C) PCR protocol 2 on a pool of 100 mosquitoes containing a single *An. stephensi* mixed with other wild-caught anophelines (*An. culicifacies*, *An. fluviatilis*, *An. minimus*, and *An. subpictus*). The numerator of numeric expression shown on the top of each lane indicates number of *An. stephensi* in a pool, and denominator indicates size of mosquito-pool. L, 100-bp ladder; NC, negative control.

**Figure 4:**
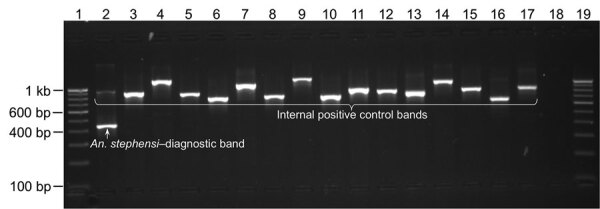
Gel photograph showing the result of PCR specific to invasive malaria vector *Anopheles stephensi* mosquitoes (protocol 1) on some individual mosquitoes belonging to the genus *Anopheles*, *Culex*, and *Aedes*. Lanes 1 and 19, 100-bp DNA ladder; 2, *An. stephensi*; 3, *An. gambiae*; 4, *An. dirus*; 5, *An. albimanus*; 6, *An. quadrimaculatus*; 7, *An. farauti;* 8, *An. freeborni*; 9, *An. funestus*; 10, *An. atroparvus*; 11, *An. merus*; 12, *An. fluviatilis* species T*;* 13, *Cx. quinquefasciatus;* 14, *An. subpictus* molecular form A; 15, *An. minimus* sensu strictu; 16, *Ae. aegypti*; 17, *Ae. albopictus*; 18, negative control.

We performed PCR protocol-1 on DNA samples isolated from individual *An. stephensi* mosquito samples, nontarget mosquitoes, and pooled samples of mixed species (<50 mosquitoes), which provided desired amplicons. All *An. stephensi* mosquitoes were positive for *An. stephensi*–specific band (438 bp**),** and all other mosquito species were negative (Table 2; Figures 3, 4). All species exhibited amplification of an IC band of varying sizes (>600 bp). *Ae. aegypti* mosquitoes exhibited the smallest IC band (≈650 bp) because of the shortest length of ITS2 (200 bp). *An. funestus* and *An. dirus* mosquitoes exhibited the largest IC bands (>1 kb) because of longer ITS2 (700 bp). PCR protocol 1 successfully identified *An. stephensi* mosquitoes in all pooled samples of mixed species, where the *An. stephensi*–specific band was prominent in pools of <20 mosquitoes. We observed that the *An. stephensi* mosquito diagnostic band grew fainter as the concentration of *An. stephensi* mosquito DNA decreased in larger pools (Figure 3, panel A). Therefore, a different protocol (protocol 2) with different primer concentrations was adopted for larger pools. PCR protocol 2 successfully identified *An. stephensi* mosquitoes in pools of 25–500 mosquitoes and provided clearly visible *An. stephensi*–specific band in pools of <100 mosquitoes. On the basis of these results, we found PCR protocol-1 suitable for individual samples or smaller pools (up to 25) (Figure 3, panel A) and PCR protocol-2 suitable for larger pools (25–100 mosquitoes) (Figure 3, panels B and C).

### Sequencing Results

*An. stephensi*–targeted DNA sequencing was successful with all 4 sequencing primers. The quality of DNA sequences generated from pooled mosquitoes was reasonably high (Appendix Figure 2). The output sequences showed 100% similarity with *An. stephensi* mosquito sequences in a BLAST search. The second highest similarity was with *An. superpictus* mosquitoes, which demonstrated 94.88% similarity on the basis of only 73% coverage. The remaining 27% nucleotide sequence belonging to the ITS2 region did not show a match with any organism other than *An. stephensi* mosquitoes.

## Discussion

The molecular methods developed in this study can identify and confirm *An. stephensi* mosquitoes individually or in a large pool of mixed mosquito species, which could enable screening of large numbers of samples collected through a variety of methods (such as light trap, pyrethrum spray collection, larval collection, etc.) with relatively limited effort and time. This method could help in early reporting of the presence of *An. stephensi* mosquitoes to concerned state health agencies and WHO.

In molecular diagnostics, we designed *An. stephensi*–specific primers and probes from a segment of ITS2 lacking homology to any other organisms for which the rDNA sequence database is available in the public domain, enabling the design of primers that are highly specific to *An. stephensi* mosquitoes and refractory to nonspecific annealing. However, this process does not preclude the possibility of any mosquito’s sequence, as-yet unreported, having matching sequences with *An. stephensi*–specific primers or probes. Therefore, confirmatory DNA sequencing should be performed in new areas using the *An. stephensi*–specific primers suggested in this report. 

In earlier studies, confirmation of this species was done through sequencing of ITS2 and mitochondrial DNA using universal primers, which cannot be used in pooled samples of mixed species. Moreover, Mishra et al. (*14*) have shown that direct DNA sequencing of ITS2 in the case of *An. stephensi* mosquitoes is not fruitful because of the presence of indel variants, which causes the collapse of a sequence starting from the indel position. The method proposed here for sequencing *An. stephensi* mosquitoes in a pooled sample was targeted to indel-free partial ITS2 (which lacks homology with any organism) and D1-D2 domains of 28S of rDNA, which are species-informative.

The real-time PCR developed in this study is for diagnostic purposes only and by no means intended for quantitative PCR (qPCR); qPCR is not reliable in the case of pooled mosquitoes belonging to different species because of interspecific variations in rDNA copy number (*16*) and body mass. However, the proportion of *An. stephensi* mosquitoes in a mosquito population, when present in extremely low density, can be obtained by the method used for the estimation of infection rates in hematophagous insects by estimating minimum infection rate or maximum-likelihood procedure (*17*) on the basis of the number of pools positive, methods frequently used for xenomonitoring.

LOD for the real-time PCR is considered a vital criterion for assessing the sensitivity of real-time PCR when the copy number of target nucleic acid is a limiting factor (e.g., detecting pathogens in an organism). However, LOD is not a limiting factor for the *An. stephensi*–specific diagnostic real-time PCR; rDNA is abundantly found in the organism because of its high copy number. We observed in this study that LOD cannot be a limiting factor even when the proportion of target mosquitoes is 1/500 in a pooled sample or tested on DNA isolated from a single leg (Ct values <24). On the basis of our observations, we suggest a cutoff value of 30 for real-time PCR for more reliable results. Ct values above this threshold can be suspected to be DNA contamination which should be verified through DNA sequencing (Appendix). In this study, we observed false positivity for *An. stephensi* mosquitoes with late Ct values (>32) in 2 DNA samples (1 each of the *Ae. aegypti* and *An. subpictus* mosquito) because of the contamination of DNA from *An. stephensi* mosquitoes.

The diagnostic PCRs in this study were designed to identify *An. stephensi* mosquitoes in large pools of samples. However, pooling of a large number of samples can accumulate potential PCR inhibitors. The heme compound in the blood (*18*) and eye pigment in the head of an insect (*19*) are reported potential inhibitors. Although we did not observe inhibitory effect of pooling in the real-time PCR, we observed substantial inhibitory effect in a size-diagnostic PCR with a pool of >50 mosquitoes. In this study, we experienced an improvement in the intensity of the band in size-diagnostic PCR in such pools by diluting DNA.

Although we have successfully demonstrated identifying a single *An. stephensi* mosquito in pools of 500 mosquitoes, using a pool of up to 100 mosquitoes that can be ground in a single microcentrifuge tube during DNA isolation without the need for grinding by mortar and pestle is recommended. Grinding by using a mortar and pestle might increase the risk for carryover contamination. For confirmation of *An. stephensi* mosquitoes in pooled samples through Sanger sequencing, we suggest using internal primers (Stq-F and StD2-R, both of which are specific to *An. stephensi* mosquitoes) for sequence termination reactions as a precautionary measure. This step is critical to rule out sequencing of false-positive PCR products because of nonspecific annealing with unknown nontarget species, if any.

In conclusion, the molecular tools developed in this study can be used to identify and confirm *An. stephensi* mosquitoes, individually or in a pool of mixed mosquito species. This process will enable health authorities to detect early invasion of the species, especially in areas where it exists with low density.

Appendix Additional information about study of molecular tools for early detection of invasive malaria vector *Anopheles stephensi* mosquitoes
