# Supplementary material for: Molecular Tools for Early Detection of Invasive Malaria Vector Anopheles stephensi Mosquitoes
Source: Emerg Infect Dis. 2023 Jan;29(1):36–44. doi: 10.3201/eid2901.220786 (PMC9796203; doi:10.3201/eid2901.220786)
Supplement: Appendix — Additional information about study of molecular tools for early detection of invasive malaria vector Anopheles stephensi mosquitoes [file 22-0786-Techapp-s1.pdf]

Article DOI: https://doi.org/10.3201/eid2901.220786

Molecular Tools for Early Detection of Invasive Malaria Vector *Anopheles stephensi* Mosquitoes

Appendix

DNA Sequencing of Mosquito Samples Detected False Positive for *Anopheles stephensi* in Real-time PCR with Late Ct Value

Two pre-isolated DNA of mosquitoes, one each of *Aedes aegypti* and *Anopheles. subpictus,* were detected false-positive as *Anopheles stephensi* by real-time PCR with late Ct values (32.95 and 33.09, respectively). To check if false positivity is due to the contamination of DNA from *An. stephensi*, these samples were subjected to DNA sequencing following methods described in the main article under section "DNA sequencing strategy for the confirmation of PCR-based identification of *An. stephensi* in pooled samples" with modification where number of PCR cycles were increased to 45. Both samples showed ≈450 bp amplicon (Appendix Figure 3). The PCR products were sequenced from both directions of the strand. NCBI-blast search revealed 100% homology of all sequences with *An. stephensi*, confirming contamination of samples with DNA from *An. stephensi*.

Appendix Table. List of biologic material used to evaluate specificity of *An. stephensi*-diagnostic PCR assays

Description of species	Source and nature of material (origin)
Anopheline species	
An. albimanus, strain STECLA	BEI Resources, USA, frozen adult (Santa Tecla, El Salvador)
An. atroparvus, strain EBRO	BEI Resources, USA, frozen adult (Tarragona, Amposta village, Ebro delta, Spain)
An. dirus, strain WRAIR2	BEI Resources, USA, frozen adult (Thailand)
An. farauti, strain FAR1	BEI Resources, USA, frozen adult (Papua, New Guinea)
An. freeborni, strain F-1	BEI Resources, USA, frozen adult (USA Marysville, California)
An. funestus, strain FUMOZ	BEI Resources, USA, frozen adult (Matolo Province, southern Mozambique)
An. gambiae, strain G3	BEI Resources, USA, frozen adult (Pimperena, Mali)
An. merus, strain OPHANSI	BEI Resources, USA, frozen adult (South Africa)
An. quadrimaculatus, strain ORLANDO	BEI Resources, USA, frozen adult (USA)
An. stephensi, strain STE2	BEI Resources, USA, frozen adult (India)
An. stephensi type form, strain DEL	Lab colony, adults and larvae (Delhi)
An. culicifacies species A, strain NIMR	Lab colony, adults (Delhi)
An. culicifacies species B	Pre-isolated DNA from adults (Raipur, India)
An. fluviatilis species S	Pre-isolated DNA from adults (Sundergarh, India)
An. fluviatilis species T	Pre-isolated DNA from adults (Hardwar, India)
An. subpictus molecular form A	Wild caught adults (Delhi, India)
An. subpictus molecular form A	Pre-isolated DNA from adults (Raipur, India)
An. subpictus molecular form B	Pre-isolated DNA from adults (Chilka, Odisha, India)
An. sundaicus cytoform D	Pre-isolated DNA from adults (A&N Island, India)
An. stephensi intermediate form	Wild caught adults (Nuh, Haryana, India)
An. stephensi var. mysorensis	Wild caught adults (Nuh, Haryana, India)
Culicines species	
Ae. aegypti, NIMR strain	Lab colony, adults (Delhi)
Ae. aegypti	Pre-isolated DNA from adults (Bengaluru)
Ae. albopictus	Wild caught adults (Delhi, India)
Cx. quinquefasciatus	Wild caught adults (Delhi, India)
Pool of mixed species (*An. culicifacies s.l., An. subpictus s.l., An. fluviatilis s.l.* and *Cx. quinquefasciatus)*	Wild caught adults (Dadri, Uttar Pradesh, India)

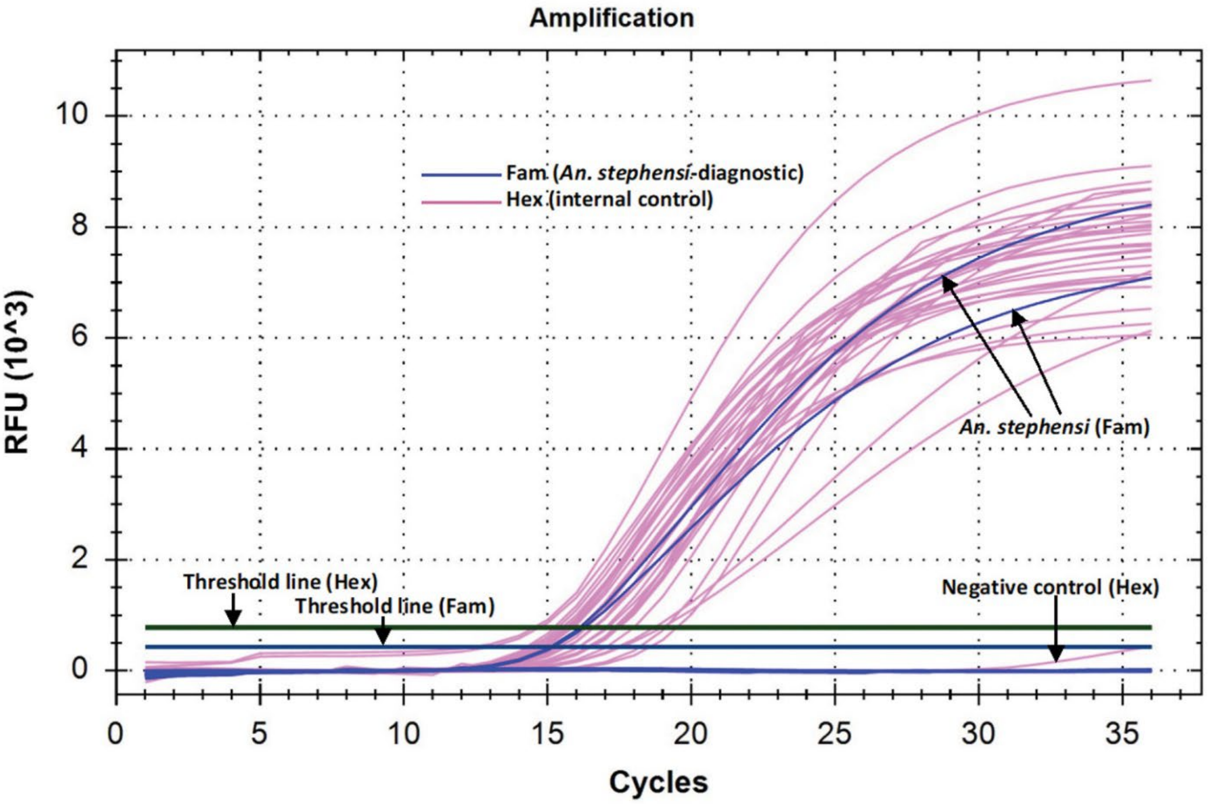

Appendix Figure 1. Amplification curve of real-time PCR on *An. stephensi* and other mosquitoes (anophelines and culicine) showing threshold lines for Fam and Hex as determine by the software. Amplification crossing threshold value for Fam was seen in *An. stephensi* only and for Hex was seen in all mosquitoes tested (*An. stephensi, An. albimanus, An. quadrimaculatus, An. dirus, An. farauti, An. gambiae, An. freeborni, An. funestus, An. atroparvus, An. culicifacies* complex, *An. subpictus* complex, *An. merus, An. fluviatilis* complex, *Cx. quinquefasciatus*).

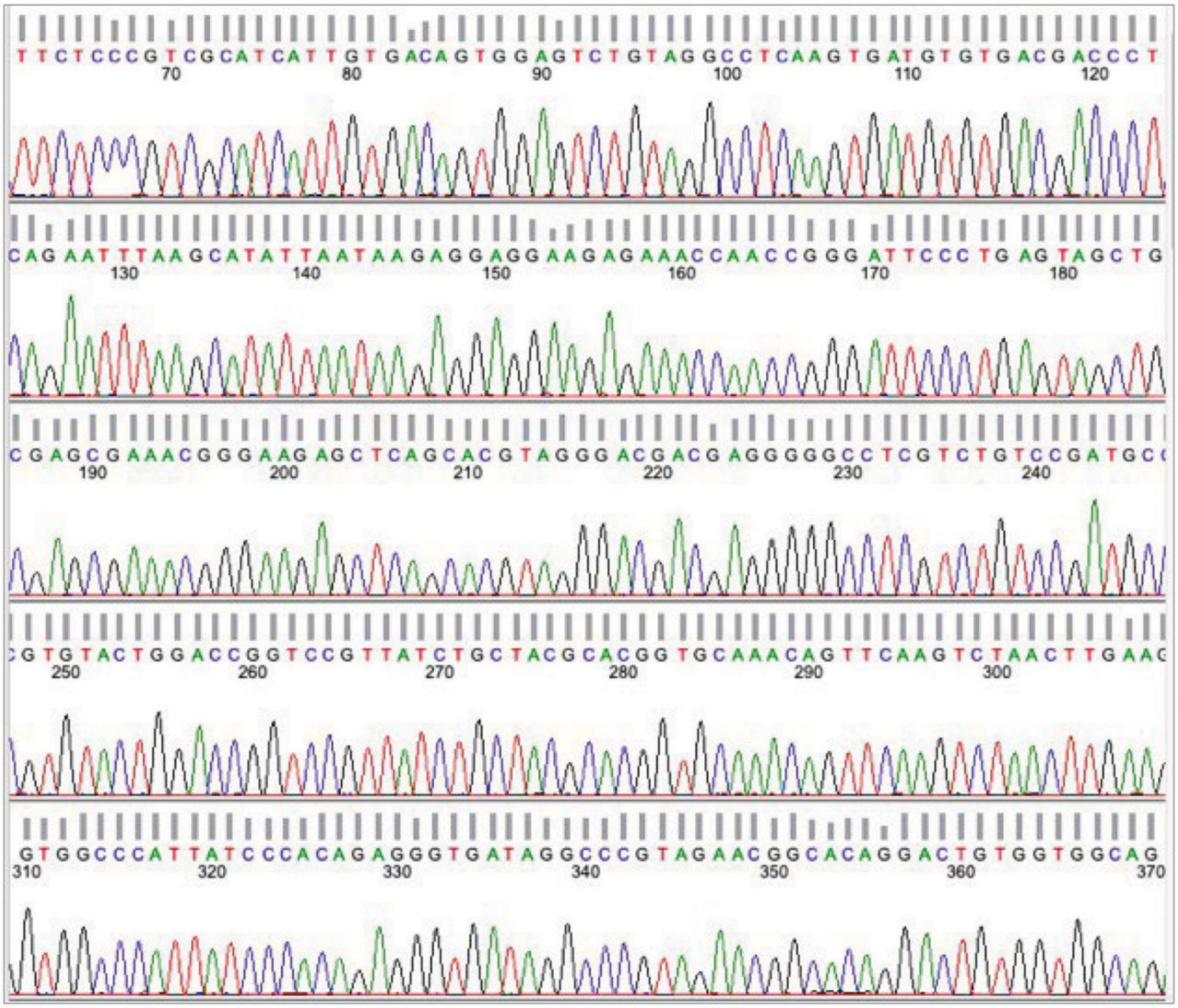

Appendix Figure 2. DNA sequence chromatogram (partial ITS2 and 28S) of *An. stephensi* showing the quality of sequence derived from DNA isolated from a pool of 500 mosquitoes containing single *An. stephensi* and rest *An. culicifacies.*

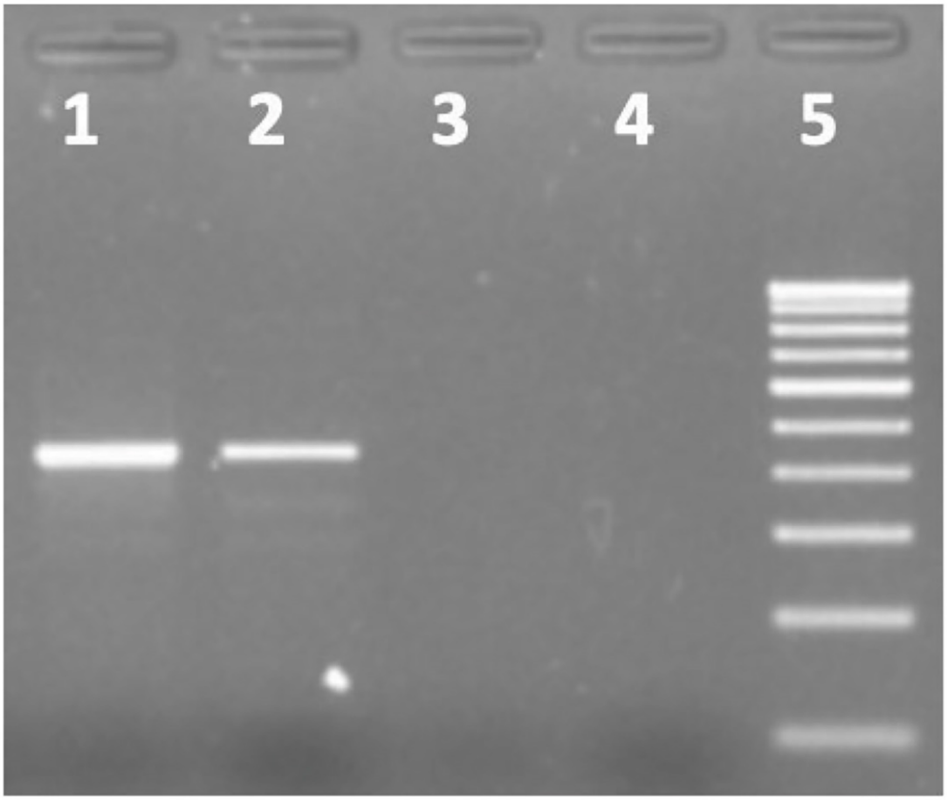

Appendix Figure 3. Gel photograph showing amplification of *An. stephensi*-specific PCR product in DNA isolated from *Ae. aegypti* (lane 1) and *An. subpictus* (lane 2) which were detected false positive for *An. stephensi* in real-time PCR with late Ct values. Lanes 3 and 4 are negative controls; Lane 5: 100 bp DNA ladder.
